# Association of peripheral immunity with cognition, neuroimaging, and Alzheimer’s pathology

**DOI:** 10.1186/s13195-022-00968-y

**Published:** 2022-02-09

**Authors:** Jia-Hui Hou, Ya-Nan Ou, Wei Xu, Peng-Fei Zhang, Lan Tan, Jin-Tai Yu, on behalf of Alzheimer’s Disease Neuroimaging Initiative

**Affiliations:** 1 Department of Neurology, Qingdao Municipal Hospital, Qingdao University, Qingdao, 266071 China; 2 Department of Neurology and Institute of Neurology, Huashan Hospital, Shanghai Medical College, Fudan University, 12th Wulumuqi Zhong Road, Shanghai, 200040 China

**Keywords:** Peripheral immunity, Alzheimer’s disease, Neutrophil-lymphocyte ratio, Neutrophil, Lymphocyte, Mediation

## Abstract

**Background:**

Neuroinflammation has been considered to be a driving force of Alzheimer’s disease. However, the association between peripheral immunity and AD has been rarely investigated.

**Methods:**

Separate regression analyses were conducted to explore the associations among peripheral immune markers and cognition, neuroimaging, and AD pathology. Causal mediation analyses were used to investigate whether the associations with cognition were mediated by AD pathology.

**Results:**

A total of 1107 participants (43.9% female, mean age of 73.2 years) from the Alzheimer’s Disease Neuroimaging Initiative (ADNI) were included. Regression analyses indicated that elevated neutrophils (NEU) count and neutrophil-lymphocyte ratio (NLR) were associated with lower levels of global cognition, memory function (MEM), and executive function (EF), and reduced brain metabolism by 18F-fluorodeoxyglucose-positron emission tomography (FDG-PET) as well as greater ventricular volume. An elevated NLR was associated with a lower level of β-amyloid (Aβ) and a higher level of total tau (T-tau) in cerebrospinal fluid (CSF), smaller hippocampal volume (HV), and lesser entorhinal cortex (EC) thickness. On the contrary, an elevated level of lymphocytes (LYM) was associated with a higher level of Aβ and a lower level of T-tau in CSF, better cognition, and less atrophy of brain regions (ventricular volume, HV, and EC thickness). The associations of LYM and NLR with cognition were mediated by Aβ and T-tau pathology (proportion: 18%~64%; *p* < 0.05).

**Conclusions:**

We revealed that two types of peripheral immune cells (NEU and LYM) and the ratio of these two cell types (NLR) had associations with cognition, neuroimaging, and AD pathology. The associations might be mediated by Aβ and tau pathology.

**Supplementary Information:**

The online version contains supplementary material available at 10.1186/s13195-022-00968-y.

## Background

Alzheimer’s disease (AD) is a common neurodegenerative disease pathologically characterized by neurofibrillary tangles and widespread senile plaques. The clinical hallmark of AD is gradual declines in memory and other cognitive functions. Drugs and other interventions are almost impossible to achieve satisfactory efficacy for advanced AD (cognitive impairment) [1]. Therefore, early detection and treatment of AD are important. The detection methods for existing well-established biomarkers, including β-amyloid (Aβ), phosphorylated-tau (P-tau), and total tau (T-tau) in cerebrospinal fluid (CSF), and techniques such as structural magnetic resonance imaging (MRI) and positron emission tomography imaging for AD are invasive, expensive, inconvenient, and difficult to implement under resource-limited settings [2]. To reduce its cost and enhance its generalizability, the identification of less-invasive and cheap indicators for AD is becoming increasingly important. Undoubtedly, peripheral blood indicators are a better choice. Furthermore, anti-inflammatory therapy might become a new direction for the treatment or prevention of dementia, even during the early stage (absence of cognitive impairment) of AD. Therefore, there is an urgent need to explore the associations of peripheral immunity with cognition, neuroimaging and AD pathology.

It has been proposed that neuroinflammation is a possible cause or driving force of AD by contributing to neurodegeneration and pathogenesis across all stages of the disease [3]. AD is a systemic disease that involves a dynamic peripheral and central immune responses [4], and growing studies have shown a pivotal contribution of the peripheral immune system. Previous studies have reported changes in the peripheral immune systems of AD patients, especially in aspects of the cell count of neutrophils (NEU) and lymphocytes (LYM) as well as neutrophil-lymphocyte ratio (NLR). As the most important component in the peripheral immune system, NEU were found to co-exist with Aβ deposition in the brain tissues of AD patients [5]. Adaptive immune cells including T and B lymphocytes also have a great influence on the inflammatory responses in the brains of AD patients [6]. NLR is a useful and cost-effective biomarker that indicates peripheral systemic inflammation [7]. Existing evidence suggests that peripheral immunity may play a pivotal part in the progression of AD [8]. However, studies exploring associations between peripheral immunity with AD in human cohorts are scarce. Herein, we explored the associations between peripheral immune markers (NEU, LYM, and NLR) and cognition, neuroimaging and AD pathology, and tried to determine whether the associations between peripheral immune markers and cognition were mediated by AD core pathology.

## Materials and methods

### Participants

Data applied in this study were acquired from the ADNI database (http://adniloni.usc.edu), which is designed to test biochemical, clinical biomarkers, genetics, and imaging of AD. Participants received systematic neuropsychological evaluations, as well as neurological and physical examinations at baseline and follow-up, and were offered biological samples such as CSF, blood, and urine throughout the study. This multisite longitudinal biomarker research program authorized by the institutional review committee at all participating locations has acquired written informed consent from participants. The study population is composed of all cognitively normal (CN), mild cognitive impairment (MCI), and AD participants with available NEU count, LYM count, NLR level, as well as data on cognition, neuroimaging, and AD pathology.

### Peripheral immune cells

Peripheral immune cells were examined in a subset of participants from ADNI-1. Blood samples were drawn by trained professionals from the venous blood in the morning after an overnight fast, and were sent for analysis on the same day. NEU count, LYM count, and NLR were analyzed using an automated system. More method details could be found at http://adni.loni.usc.edu.

### CSF measurements

CSF was sampled by lumbar puncture, with CSF Aβ, CSF T-tau, and CSF P-tau measured at the ADNI Biomarker Core Laboratory (University of Pennsylvania) using a complex platform (xMAP; Luminex Corporation) with Innogenetics (INNO-BIA AlzBio3; Ghent, Belgium; for research use only reagents) immune assay kit-based reagents and analyzed on an automatic Elecsys cobas e 601 instrument (F. Hoffmann-La Roche) by an advanced technology known as electrochemiluminescence immunoassays (Elecsys; Roche Diagnostics, F. Hoffmann-La Roche, Basel, Switzerland).

### Cognition

Cognitive functions were assessed by several scales. Precisely, global cognition was evaluated by the Mini-Mental State Examination (MMSE), the Alzheimer’s Disease Assessment Scale (ADAS), and the Clinical Dementia Rating Sum of Boxes (CDRSB). And cognitive domains were evaluated by inspecting the neuropsychological batteries to confirm elements that can be indicators considered of memory function (MEM) and executive function (EF) [9, 10]. All assessments were carried out at baseline and follow-up.

### Neuroimaging

The 18F-fluorodeoxyglucose-positron emission tomography (FDG-PET) data were obtained and rebuilt following a standardized process (http://adni.loni.ucla.edu/). Spatial normalization of every participant’s positron emission tomography image to the standard template was implemented applying SPM529. We averaged the FDG-PET counts in temporal, angular, and posterior cingulate regions. Structural brain images were obtained using a 1.5-T MRI imaging system with T1-weighted MRI scans using a sagittal volumetric magnetization-prepared rapid acquisition gradient-echo sequence. Cortical thickness and subcortical volumes were quantified by a software program (https://surfer.nmr.mgh.harvard.edu/).

### Statistical analysis

On the basis of the cut-off threshold of CSF Aβ, the population was divided into Aβ+ (concentration levels ≤ 192 pg/ml) and Aβ− (> 192 pg/ml) groups. This further resulted in five combinations of biomarkers, consisting of CN Aβ− (CN−), CN Aβ+ (CN+), MCI Aβ− (MCI−), MCI Aβ+ (MCI+), and AD Aβ+ (AD+) after excluding 15 AD Aβ− (AD−) subjects [11]. Chi-square analysis and non-parametric analysis were used to test the inter-group differences. Categorical variables were expressed as number (percentage), and continuous variables were expressed as mean ± standard deviation (SD).

First, we separately assessed the associations of various peripheral immune markers (independent variables) with AD pathology (CSF biomarkers), cognition (global cognition, as well as MEM and EF), and neuroimaging (brain structure and FDG-PET) using multiple linear regression models and linear mixed-effects models. Extreme values of more than 3 SDs from the mean were excluded before the independent variables were normalized by log-transformation. Moreover, we added the interaction analysis between peripheral immune markers with *APOE* ε4 status and gender into multiple linear regression models, to determine whether the associations of peripheral immunity with AD pathology, cognition, and neuroimaging independent of *APOE* ε4 status and gender as modifiers, and to determine whether strata effects existed.

Next, the mediation analyses were conducted via “mediate,” “car,” and “lm” packages in R software (version 4.0.3) to explore whether the associations between peripheral immune markers and cognition were mediated by AD core pathology. Linear regression models were fitted on the basis of the methods proposed by Baron and Kenny [12]. The first equation showed the effect of the independent variable on mediators. The second equation showed the effect of mediators on dependent variables after controlling the influence of independent variables. The third equation showed the total effect of independent variables on dependent variables, the direct effect of independent variables on dependent variables after controlling the influence of mediators, and the indirect effect of independent variables on dependent variables without controlling the influence of mediators.

Covariates in all the correlation analyses included gender, age, *APOE* ε4 status, and education level, and intracranial volume was added as a covariate when dependent variables were associated with brain structure. A two-tailed *p* < 0.05 was considered significant. The R software (version 4.0.3), GraphPad Prism version 7.00 (San Diego, CA), and IBM SPSS Statistics 26 were applied for figure preparation and statistical analyses.

## Results

### Characteristics of participants

The present analysis included 1107 participants, consisting of 168 CN−, 123 CN+, 217 MCI−, 403 MCI+, and 196 AD+ participants. The whole population had a female proportion of 43.9%, an age range from 54 to 91 years old (73.20 ± 7.29 years old), and an *APOE* ε4 positive percentage of 47.97% (Table 1). Among them, 1088 participants were followed up with longitudinal data (see Additional file 1). Except for gender, basic demographics, biomarker levels, cognitive scores, brain structure, and levels of peripheral immune markers all illustrated statistically significant intergroup differences (*p* < 0.05).

**Table 1:** Basic characteristics of population included

Characteristics	CN-	CN+	MCI-	MCI+	AD+	***p***
*Number*	168	123	217	403	196	
Age (years)	73.41 ± 5.82	75.34 ± 5.89	70.78 ± 8.01	73.21 ± 7.14	74.35 ± 8.00	**< 0.001**
Female gender (%)	81 (48.21)	65 (52.85)	94 (43.32)	163 (40.45)	83 (42.35)	0.114
Education (years)	16.40 ± 2.65	16.17 ± 2.66	16.15 ± 2.69	16.01 ± 2.81	15.47 ± 2.97	**0.042**
*APOE ε4* carriers (%)	22 (13.10)	54 (43.90)	44 (20.28)	263 (65.26)	148 (75.51)	**< 0.001**
*Biomarkers*						
CSF Aβ (pg/ml)	237.65 ± 25.07	147.55 ± 25.88	234.05 ± 25.86	138.18 ± 24.93	129.95 ± 22.01	**< 0.001**
CSF P-tau (pg/ml)	26.27 ± 12.25	35.48 ± 18.43	24.81 ± 11.64	45.09 ± 19.58	47.02 ± 18.95	**< 0.001**
CSF T-tau (pg/ml)	59.76 ± 23.74	75.96 ± 37.86	57.00 ± 25.10	103.7 ± 48.62	122.39 ± 49.86	**< 0.001**
FDG-PET	1.33 ± 0.12	1.30 ± 0.13	1.30 ± 0.11	1.22 ± 0.13	1.06 ± 0.13	**< 0.001**
*Cognitive scores*						
MMSE	29.01 ± 1.25	29.04 ± 1.14	28.27 ± 1.60	27.41 ± 1.86	23.24 ± 1.95	**< 0.001**
CDRSB	0.03 ± 0.12	0.03 ± 0.14	1.25 ± 0.76	1.61 ± 0.92	4.49 ± 1.60	**< 0.001**
ADAS	9.09 ± 4.38	9.74 ± 4.38	12.93 ± 5.84	17.41 ± 6.79	30.43 ± 8.20	**< 0.001**
MEM	1.16 ± 0.53	0.95 + 0.59	0.48 ± 0.69	-0.06 ± 0.56	-0.87 ± 0.50	**< 0.001**
EF	0.94 ± 0.79	0.54 ± 0.73	0.53 ± 0.89	0.01 ± 0.82	-0.91 ± 0.87	**< 0.001**
*Brain structure*						
HV (mm^3^)	7446.36 ± 908.34	7363.18 ± 785.76	7293.58 ± 1154.07	6633.39 ± 1058.85	5854.03 ± 1013.51	**< 0.001**
EC thickness (mm)	3845.57 ± 635.32	3855.90 ± 547.01	3737.91 ± 684.43	3454.81 ± 733.31	2833.39 ± 661.89	**< 0.001**
Ventricular volume (mm^3^)	30620.87 ± 15303.06	37625.13 ± 19034.18	34734.47 ± 19635.79	40983.89 ± 21601.68	47828.71 ± 21097.12	**< 0.001**
*Peripheral immune cells*						
NEU (× 10^3^/μL)	3.77 ± 1.89	4.01 ± 1.17	3.87 ± 1.08	4.10 ± 1.19	4.28 ± 1.20	**< 0.001**
LYM (×10^3^/μL)	1.89 ± 0.53	1.74 ± 0.47	1.87 ± 0.58	1.71 ± 0.53	1.70 ± 0.52	**< 0.001**
NLR (×10^3^/μL)	2.14 ± 0.93	2.41 ± 0.89	2.23 ± 0.92	2.50 ± 0.96	2.71 ± 1.06	**< 0.001**

*Abbreviations*: *CN* cognitively normal, *MCI *mild cognitive impairment, *AD *Alzheimer’s disease, *CSF *cerebrospinal fluid, *Aβ *β-amyloid, *P-tau *phosphorylated-tau, *T-tau *total tau, *FDG-PET *18F-fluorodeoxyglucose-positron emission tomography, *MMSE *Mini-Mental State, *CDRSB *Clinical Dementia Rating Sum of Boxes, *ADAS *Alzheimer’s Disease Assessment Scale, *MEM *memory function, *EF *executive function, *HV *hippocampal volume, *EC *entorhinal cortex, *NEU *neutrophils, *LYM *lymphocytes, *NLR *neutrophil–lymphocyte ratio; values are mean ± standard deviation (SD), or *n* (% of the group)

**Fig. 1:**
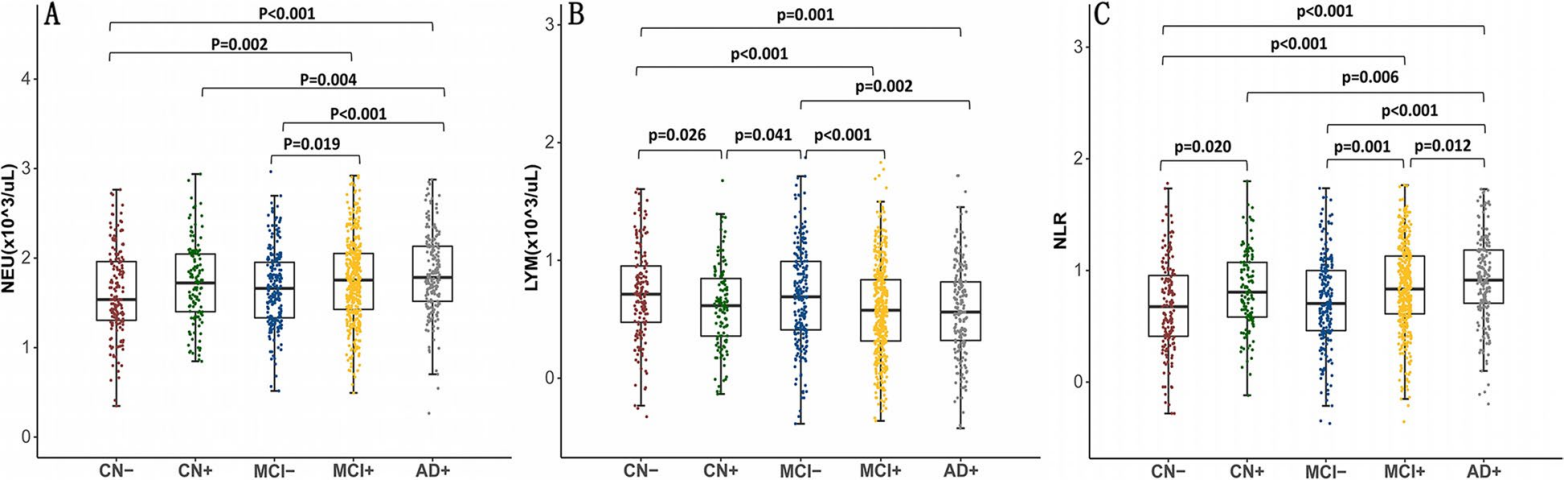
The intergroup differences in peripheral immune markers among different groups at baseline. CN, cognitively normal; MCI, mild cognitive impairment; AD, Alzheimer’s disease; NEU: neutrophils; LYM: lymphocytes; NLR: neutrophil-lymphocyte ratio

As shown in Fig. 1, baseline peripheral NEU count was significantly higher in the AD+ group than in the MCI− (*p* < 0.001), and CN+/− (both *p* < 0.01) groups, and the count was also higher in the MCI+ group than those in the MCI- (*p* = 0.019) and CN− (*p* = 0.002) groups. Baseline peripheral NLR level was significantly higher in the AD+ group than in the MCI+/− (both *p* < 0.05), and CN+/− (both *p* < 0.01) groups. The level was also higher in the MCI+ group compared with those in the MCI− (*p* = 0.001) and CN− (*p* < 0.001) groups. And the level in CN+ group is higher than that in CN− (*p* = 0.020) group. Baseline peripheral LYM count was significantly lower in the AD+ group than those in MCI− (*p* = 0.002) and CN− (*p* = 0.001) groups. The count was also lower in the MCI+ group compared with MCI− (*p* < 0.001) and CN− (*p* < 0.001) groups. The count in CN− group is lower than in CN+ (*p* = 0.026) group, and the count in MCI− is lower than that in CN+ (*p* = 0.041) group.

### Baseline associations of peripheral immunity with cognition, neuroimaging, and AD pathology

**Fig. 2:**
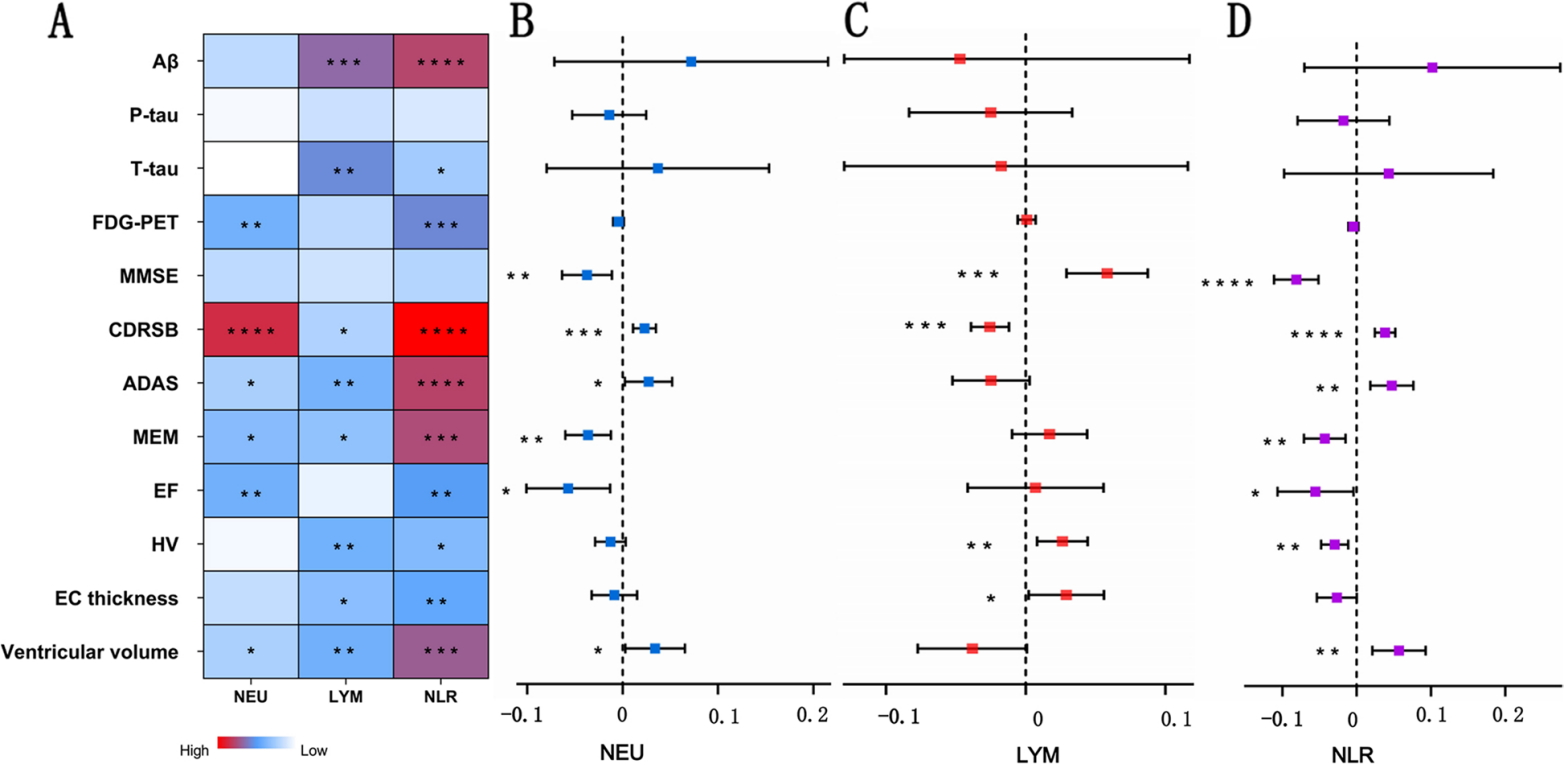
Linear correlation between peripheral immunity with cognition, neuroimaging and AD pathology. CN, cognitively normal; MCI, mild cognitive impairment; AD, Alzheimer’s disease; CSF, cerebrospinal fluid; Aβ, β-Amyloid; P-tau, phosphorylated-tau; T-tau, total tau; FDG-PET:18F-fluorodeoxyglucose-positron emission tomography; MMSE, Mini-Mental State; CDRSB, Clinical Dementia Rating Sum of Boxes; ADAS, Alzheimer’s Disease Assessment Scale; MEM, memory function; EF, executive function; HV, hippocampal volume; EC, entorhinal cortex; NEU, neutrophils; LYM, lymphocytes; NLR, neutrophil-lymphocyte ratio; 0.01 ≤ * ≤ 0.05, 0.001 ≤ ** < 0.01, 0.0001 ≤ *** < 0.001, **** < 0.0001

Without considering diagnostic category, findings in the general population at baseline were shown in Fig. 2A and Additional file 2. Elevated NEU count was associated with reduced brain metabolism (*β* = −0.035, *p* = 0.006 for FDG-PET), lower levels of global cognition (*β* = 0.121, *p* < 0.001 for CDRSB, *β* = 0.172, *p* = 0.041 for ADAS), MEM (*β* = −0.177, *p* = 0.012) and EF (*β* = −0.231, *p* = 0.006), as well as greater ventricular volume (*β* = 0.416, *p* = 0.042). Elevated level of NLR was associated with a lower level of Aβ (*β* = −0.078, *p* < 0.001) and a higher level of T-tau (*β* = 0.080, *p* = 0.033) in CSF, reduced brain metabolism (*β* = −0.050, *p* = 0.001 for FDG-PET), lower levels of global cognition (*β* = 0.153, *p* < 0.001 for CDRSB, and *β* = 0.391, *p* < 0.001 for ADAS), MEM (*β* = −0.322, *p* < 0.001) and EF (*β* = −0.306, *p* = 0.002), smaller hippocampal volume (HV) (*β* = −0.184, *p* = 0.011), and lesser entorhinal cortex (EC) thickness (*β* = −0.217, *p* = 0.004), as well as greater ventricular volume (*β* = 0.889, *p* < 0.001). However, elevated LYM count was associated with a higher level of Aβ (*β* = 0.071, *p*< 0.001) and a lower level of T-tau (*β* = −0.124, *p* = 0.001) in CSF, better levels of global cognition (*β* = −0.066 *p* = 0.051 for CDRSB, and *β* = −0.263, *p* = 0.007 for ADAS) and MEM (*β* = 0.198, *p* = 0.018), greater HV (*β* = 0.200, *p* = 0.006) and EC thickness (*β* = 0.185, *p* = 0.014), as well as smaller ventricular volume (*β* = −0.652, *p* = 0.006).

Taking diagnostic categories into consideration barely changed the identified associations of peripheral immunity with cognition, neuroimaging, and AD pathology (see Additional files 4, 5 and 6).

### Associations of baseline peripheral immunity with longitudinal changes in cognition, neuroimaging, and AD pathology

Regardless of diagnosis category, as shown in Fig. 2B–D and Additional file 3, higher NEU count and higher NLR level were associated with a faster cognitive decline as indicated by changes in MMSE score (estimate = − 0.037, *p* = 0.005 for NEU, and estimate = − 0.081, *p* < 0.001 for NLR), CDRSB score (estimate = 0.023, *p* < 0.001 for NEU, and estimate = 0.038, *p* < 0.001 for NLR), ADAS score (estimate = 0.027, *p* = 0.032 for NEU, and estimate = 0.047, *p* = 0.001 for NLR), MEM score (estimate = − 0.036, *p* = 0.003 for NEU, and estimate = − 0.043, *p* = 0.003 for NLR), EF score (estimate = − 0.057, *p* = 0.011 for NEU, and estimate = − 0.055, *p* = 0.034 for NLR), and ventricular volume (estimate = 0.034, *p* = 0.032 for NEU, and estimate = 0.057, *p* = 0.002 for NLR), higher NLR level was associated with a faster cognitive decline as indicated by changes in HV (estimate = − 0.030, *p* = 0.002). Besides, higher LYM count was associated with a slower cognitive decline as indicated by changes in MMSE score (estimate = 0.058, *p* < 0.001), CDRSB score (estimate = − 0.026, *p* < 0.001), HV (estimate = 0.026, *p* = 0.005), and EC thickness (estimate = 0.029, *p* = 0.036).

Moreover, taking diagnostic categories into consideration, statistically significant longitudinal correlations were still found between peripheral immunity with cognition, neuroimaging, and AD pathology in each group (see Additional files 7, 8 and 9). The directions of the above longitudinal correlations were consistent with the directions of the cross-sectional correlations, indicated by regression coefficients.

### Interaction analyses

We found that the interaction between NLR and gender was obviously associated with HV (*p* = 0.024). Subgroup analyses showed that significant associations between NLR and HV were found only in female subgroup (*β* = − 0.346, *p*< 0.001), but not in male subgroup (*β* = − 0.033, *p* = 0.740). No interaction was found of peripheral immune markers with *APOE* ε4 status (see Additional file 10).

### Causal mediation analyses

**Fig. 3:**
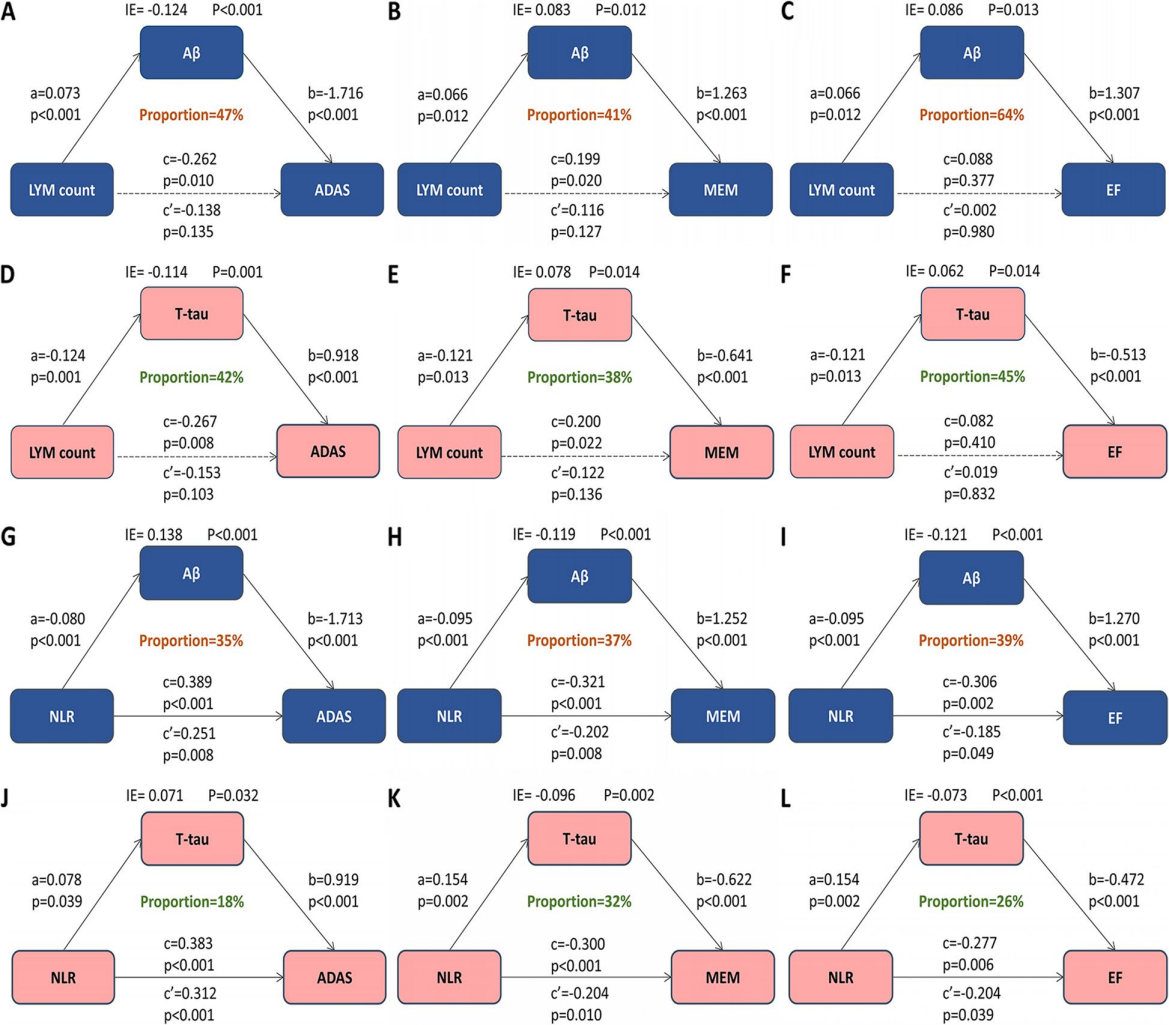
Mediation analyses with global cognition measured by ADAS, memory function, executive function as cognitive outcomes. a is the effect of independent variable on mediators; b is the effect of mediators on dependent variables after controlling the influence of independent variables; c is the total effect of independent variables on dependent variables; c’ is the direct effect of independent variables on dependent variables after controlling the influence of mediators; IE is the indirect effect of independent variables on dependent variables, in this intermediary model, c = c’ + ab. ADAS, Alzheimer's Disease Assessment Scale; MEM, memory function; EF, executive function, NEU, neutrophils; LYM, lymphocytes; NLR, neutrophil-lymphocyte ratio

The cross-sectional analysis indicated that CSF Aβ and T-tau were not only important biomarkers for cognitive impairment but also potential modulators of cognition. The indirect and total effects of LYM count on cognition, including global cognition measured by ADAS (Fig. 3A, D) as well as MEM (Fig. 3B, E), and EF (Fig. 3C, F) reached statistical significance (*p* < 0.05), but the direct effects did not (*p* > 0.05), indicating that all the associations of LYM with global cognition, MEM, and EF were completely mediated by Aβ and T-tau, with the ratio of mediation ranging from 38 to 64%. The total effects of LYM count on EF did not reach statistical significance, which might be owing to the masking effect [13]. The direct effects of NLR on cognition, including global cognition (Fig. 3D, J), MEM (Fig. 3E, K) and EF (Fig. 3E, L), were significantly lower than the indirect effects, indicating all the associations of NLR with MEM, EF and global cognition were partially mediated by Aβ and T-tau, with the ratio of mediation ranging from 18 to 39%.

## Discussion

This is a timely study that systematically explored the associations of peripheral immunity with cognition, neuroimaging and AD pathology, and explored the mediation effects of AD pathology on cognition. Precisely, our study showed two types of peripheral immune cells (NEU and LYM), and the ratio of these two cell types (NLR) were associated with AD pathology (CSF Aβ, T-tau), brain metabolism (FDG-PET), global cognition (MMSE, CDRSB and ADAS scores), MEM, EF, and neuroimaging of AD (HV, EC thickness and ventricular volume) at baseline and follow-up, and the pathological effects of these peripheral immune markers were different. Furthermore, the associations among peripheral immunity and AD pathology, cognition, and neuroimaging were independent of *APOE* ε4 status, and Aβ and T-tau pathology could mediate the influences of LYM count and NLR on cognition.

Although the detailed roles the immune system plays in AD are not fully understood and controversial, existing studies have shown a direct communication between the peripheral and central immune system [4, 14], and the changes observed in the peripheral blood are a reflection of the immune response in the brain mediated by proinflammatory cytokines that are released to the periphery. A recent study showed that a history of infections requiring hospitalization was associated with future development of AD [15], and existing meta-analysis found that non-steroidal anti-inflammatory drug users had a lower risk of developing AD compared with nonusers [16].

Neurofibrillary tangles and widespread senile plaques in the AD brain provide a significant stimulus for inflammation. Increased serum levels of tumor necrosis factor (TNF), ICAM-1, IL-1, IL-6, and other already reported pro-inflammatory cytokines in AD patients [17–22] can cause a neutrophil “alert” activation status, which is reflected by a significantly increased of NEU CD11b and Mac-1 in blood [23]. Tiffany et al. discovered that Aβ was a formyl-peptide receptor 2 agonist, indicating that Aβ was a potent chemoattractant for phagocytic leukocytes [24]. Proinflammatory cytokines contribute to neuronal dysfunction and cell death [25], and those released to the periphery can further cause changes in peripheral NEU count. Neurovasculature not only allows NEU to enter the central nervous system (CNS) but also promotes the accumulation of NEU in the CNS during neuroinflammation [26]. Once NEU are in proximity of Aβ plaques, they are activated and subsequently secrete detrimental mediators, including reactive oxygen species (ROS) [27], which further increases the permeability of the blood brain barrier (BBB) by damaging tight junctions [28]. Joseph Park et al. found that NEU were involved in Aβ-activated microglia-mediated AD pathology to enhance neuroinflammation [29]. Increased senescent NEU may act through enhanced tissue tropism and the damaged BBB to migrate to the amyloid plaque sites and release NETs, leading to aggravation of Aβ pathology [18, 26, 30]. Therefore, a vicious positive feedback cycle that can explain the associations between NEU and AD is proposed: upregulated senescent NEU that overexpress CD83 and TAP1 stimulate T cells by antigen presentation, and activated T cells in turn release proinflammatory markers which elevate the number of senescent NEU [26]. Yuan Dong et al. found that NEU phenotype might be associated with the rate of cognitive decline [28]. Kritleen K et al. found that NEU-related inflammatory factor could predict the decline in EF [31]. All the above evidences suggest that increased count of peripheral NEU has an adverse effect on cognitive function, which is consistent with our results.

With respect to LYM, the issue becomes even more complicated because there is currently no consensus on the modifications of LYM in AD. Our results are consistent with several studies that reported a reduction in the number of peripheral LYM in AD patients [32–37], which supports the hypothesis of a general decline in immune activity and cell cycle dysregulation in AD that creates a permissive environment to the pathophysiological processes happening in the brain. Cell cycle dysregulation is systemic, affecting not only neurons [38] but also peripheral LYM. Adaptive immune cells including T and B lymphocytes play a major part in the inflammatory reaction in the brains of the AD patients. Compared with healthy controls, differentiated CD3+ T-cells is increased in AD hippocampal parenchyma [6], and activated T cells generate interferon gamma (IFN-α) that can result in the deposition of Aβ, cognitive impairment, and subsequently AD [39]. During the inflammatory response, BBB is destroyed in the brains of AD patients, LYM in the peripheral blood migrate to and infiltrate into the brain, and LYM count number increases especially in the hippocampus and temporal cortex [40]. Microglia will recruit LYM from the peripheral circulation across the broken BBB into the CNS by releasing TNF- α, thus decreasing the amount of LYM in the peripheral circulation [41]. LYM in the peripheral blood migrate to the brain, infiltrate into the brain tissue [40], and facilitate the interaction between the CNS and immune system [42]. Gate et al. detected CD8+ T cells in AD-affected hippocampus and found that CD8+ T cells were adjacent to Aβ plaques [43]. In AD patients, LYM are more sensitive to ROS than in healthy controls [44]. As was mentioned, the increase of NEU count in patients with AD could lead to an increase in the release of ROS, which might be part of the reason for the observed decrease in peripheral LYM. Moreover, specific mutations relevant to AD including presenilin 1 mutation might cause the circulating LYM in AD brains more susceptible to cell death [45]. All the above evidence showed an inverse correlation between the increased count of peripheral LYM and AD diagnosis.

Moreover, we discovered that the ratio of the above two types of peripheral immune cells known as NLR was also an adverse indicator for AD, which has also been used in several other disorders, such as cardiovascular disease [46], diabetes [47], colorectal cancer [48], lung cancer [49], and Parkinson’s disease [37, 50]. Our study was consistent with these previous studies which observed a dramatic increase of peripheral NLR in AD patients compared to healthy controls [7, 51], suggesting that higher peripheral NLR has an adverse effect on cognitive function.

Prior studies showed that Aβ might be sufficient to cause cortical amyloid deposition [52] and neurodegeneration which ultimately led to cognitive deterioration [53], while T-tau probably was associated with the intensity of neuronal injury [54] and neurodegeneration [52]. Both of them had high accuracy in AD diagnosis. Our mediation analysis indicated that Aβ and T-tau might fully mediate the influences of NLR on global cognition, MEM, and EF, while Aβ and T-tau might partially mediate the influences of LYM on global cognition, MEM, and EF. Thus, it could be reasonably inferred that LYM count and NLR led to AD by contributing to cortical amyloid deposition and neuronal injury, or the causal relationship between LYM count and NLR with Aβ and T-tau might be bidirectional. These findings indicated the close associations of LYM count and NLR with cognition.

Historically, CSF and imaging biomarkers have been considered as the indicators tightly related to AD. However, the invasiveness of lumbar punctures and the cost of imaging examination limited their application. Blood-based biomarkers such as peripheral immune cells have advantages of simple operation, low cost, easy acceptability, and potential global applicability [55]. Thus, it is necessary to explore the associations of peripheral immune markers with cognition, CSF, and imaging biomarkers of AD.

According to our understanding, the topic about the association between peripheral immunity and AD is still in its infancy. Previous articles have investigated the association of peripheral immunity with AD, but they were limited to basic research [5, 6], animal research [18, 19, 30], review [26, 40, 42], and rough cross-sectional analyses [7], and most studies were of small samples [28, 33, 36, 44, 51, 56, 57]. Our study is the first original study using linear models to systematically examine the linear regression associations of peripheral immunity with cognition, neuroimaging, and AD pathology in a huge human cohort, the first study to explore whether the associations of peripheral immunity with cognition were mediated by AD core pathology, and the first study to explore the interaction among peripheral immunity and *APOE* ε4 status and gender.

In our study, patients with established AD displayed differences in NEU count, LYM count, and NLR level compared to MCI and CN groups. These intergroup differences as well as significant associations found in cross-sectional analysis indicated a positive correlation between the diagnosis of AD and higher peripheral NEU count and NLR level, while an inverse correlation between higher peripheral LYM counts and AD diagnosis. Longitudinal analysis indicated that higher peripheral NEU count and NLR level were associated with a faster cognitive decline, while higher peripheral LYM count was associated with a slower cognitive decline, which is specifically manifested in the changes of cognition scores and neuroimaging and Alzheimer's pathology. Aβ and T-tau are tightly related to AD, and the mediating effect in this study suggested that Aβ and T-tau also acted as mediators of cognition. Therefore, peripheral LYM and NLR may affect AD through cerebral amyloid deposition and neuronal injury, or the causal relationship between peripheral immune markers (LYM count and NLR) with cerebral amyloid deposition and neuronal injury might be bidirectional, thereby raising the credibility that peripheral immunity is associated with AD. For interaction effects, no interaction between peripheral immunity and *APOE* ε4 status was found in this study, which showed associations among peripheral immunity and AD pathology, cognition, and neuroimaging were independent of *APOE* ε4 status. However, gender strata effects existed between NLR and HV, and significant associations were only found in female subgroup, not in male subgroup, which showed the association between NLR and HV was dependent on gender. This may be because testosterone in males exerted its influence to an extent that HV was genetically and environmentally affected [36]. In summary, we found that peripheral immunity was associated with cognition, neuroimaging, and Alzheimer’s pathology, which can provide ideas and support for future research in this field. If the associations can be replicated in more human cohorts in the future, blood-based biomarkers may replace CSF and imaging biomarkers in the future, and anti-inflammatory therapy may become a new direction in the treatment and prevention of AD.

### Limitations

There are limitations in this study. First, our study used NEU, LYM, and NLR as indicators to reflect peripheral immune system, and other peripheral immune markers except the three should be included in future research since peripheral immune system is an intricate system. Second, the generalizability of our consequences might be limited by the study populations sources from ADNI, no longitudinal data of peripheral immune markers (NEU, LYM, and NLR) and lymphocyte subpopulations (T/B lymphocytes) were found in the ADNI cohort, which requires large-scale studies with high-quality to further analyze longitudinal correlation in the future. Third, we did not discuss the direct associations between peripheral immune markers and inflammatory markers in the nervous system. In the future, we can explore whether neuroinflammation plays a mediating role in cognition.

## Conclusions

In summary, our study found that two types of peripheral immune cells (NEU and LYM) and the ratio of these two cell types (NLR) were associated with cognition, neuroimaging, and AD pathology; the associations of LYM count and NLR level with cognition were mediated by AD core pathology. Peripheral immune markers may replace CSF and imaging biomarkers and may provide a measure for initial screening and provide new insights into the prevention and treatment of AD.

## Supplementary Information

**Additional file 1.** Study flow diagram.

**Additional file 2.** Cross-sectional associations of peripheral immunity with cognition, neuroimaging and AD pathology in all participants.

**Additional file 3.** Longitudinal associations of peripheral immunity with cognition, neuroimaging and AD pathology in all participants.

**Additional file 4.** Cross-sectional associations of peripheral immunity with cognition, neuroimaging and AD pathology in CN group.

**Additional file 5.** Cross-sectional associations of peripheral immunity with cognition, neuroimaging and AD pathology in MCI group.

**Additional file 6.** Cross-sectional associations of peripheral immunity with cognition, neuroimaging and AD pathology in AD group.

**Additional file 7.** Longitudinal associations of peripheral immunity with cognition, neuroimaging and AD pathology in CN group.

**Additional file 8.** Longitudinal associations of peripheral immunity with cognition, neuroimaging and AD pathology in MCI group.

**Additional file 9.** Longitudinal associations of peripheral immunity with cognition, neuroimaging and AD pathology in AD group.

**Additional file 10.** Interaction analysis.

## Data Availability

The dataset generated and analyzed in the current study is available from the corresponding author on reasonable request.

## Abbreviations

AD: Alzheimer’s disease
ADNI: Alzheimer’s Disease Neuroimaging Initiative
CN: Cognitively normal
MCI: Mild cognitive impairment
CSF: Cerebrospinal fluid
Aβ: β-Amyloid
P-tau: Phosphorylated-tau
T-tau: Total tau
FDG-PET: 18F-fluorodeoxyglucose-positron emission tomography
MMSE: Mini-Mental State
CDRSB: Clinical Dementia Rating Sum of Boxes
ADAS: Alzheimer's Disease Assessment Scale
MEM: Memory function
EF: Executive function
HV: Hippocampal volume
EC: Entorhinal cortex
NEU: Neutrophils
LYM: Lymphocytes
NLR: Neutrophil-lymphocyte ratio
CNS: Central nervous system
SD: Standard deviation
MRI: Magnetic resonance imaging
TNF: Tumor necrosis factor
ROS: Reactive oxygen species
BBB: Blood brain barrier
