# Supplementary figures and images for: Association of peripheral immunity with cognition, neuroimaging, and Alzheimer’s pathology
Source: Alzheimers Res Ther. 2022 Feb 9;14:29. doi: 10.1186/s13195-022-00968-y (PMC8830026; doi:10.1186/s13195-022-00968-y)

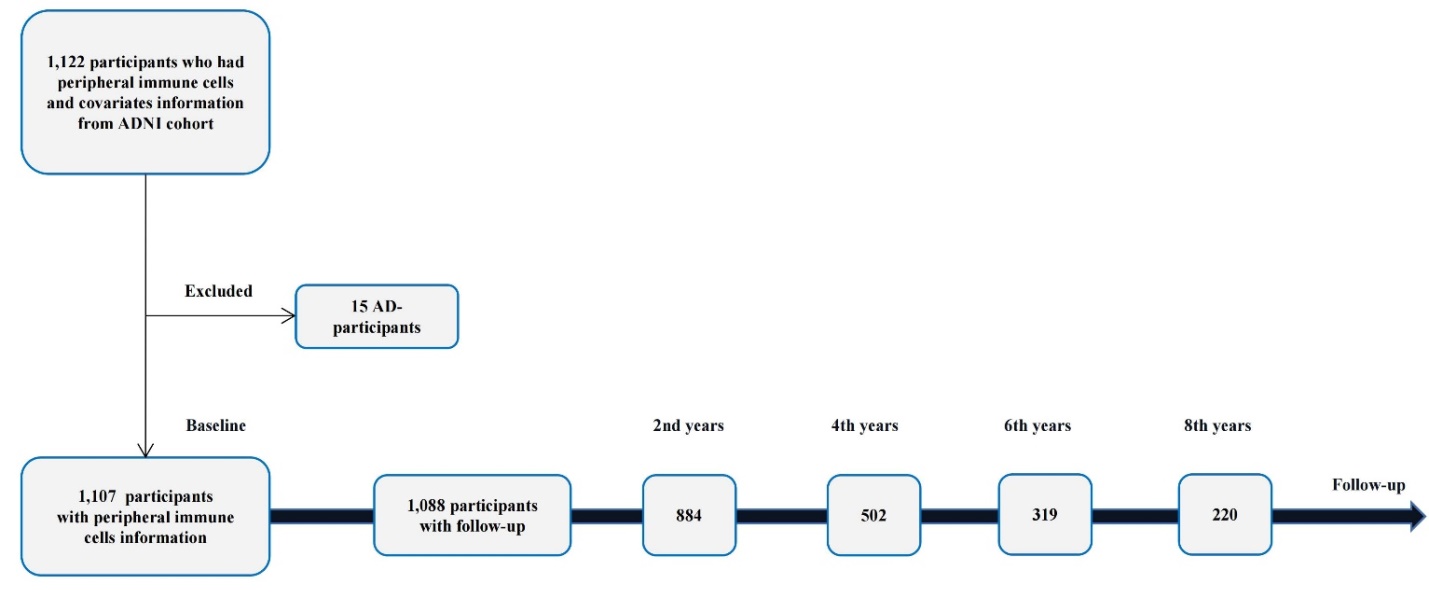

Supplement: Supplementary file 1 — Additional file 1. Study flow diagram. [file 13195_2022_968_MOESM1_ESM.docx]
