# Supplementary material for: Association of peripheral immunity with cognition, neuroimaging, and Alzheimer’s pathology
Source: Alzheimers Res Ther. 2022 Feb 9;14:29. doi: 10.1186/s13195-022-00968-y (PMC8830026; doi:10.1186/s13195-022-00968-y)
Supplement: Supplementary file 2 — Additional file 2. Cross-sectional associations of peripheral immunity with cognition, neuroimaging and AD pathology in all participants. [file 13195_2022_968_MOESM2_ESM.docx]

| Variables | NEU | | LYM | | NLR | |
| --- | --- | --- | --- | --- | --- | --- |
|  | β | P | β | P | β | P |
| Aβ | -0.029 | 0.086 | 0.071 | **< 0.001** | -0.078 | **< 0.001** |
| P-tau | 0.018 | 0.592 | -0.058 | 0.126 | 0.049 | 0.206 |
| T-tau | -0.007 | 0.827 | -0.124 | **0.001** | 0.080 | **0.033** |
| FDG-PET | -0.035 | **0.006** | 0.026 | 0.081 | -0.050 | **0.001** |
| MMSE | -0.106 | 0.085 | 0.103 | 0.149 | -0.135 | 0.060 |
| CDRSB | 0.121 | **< 0.001** | -0.066 | **0.051** | 0.153 | **< 0.001** |
| ADAS | 0.172 | **0.041** | -0.263 | **0.007** | 0.391 | **< 0.001** |
| MEM | -0.177 | **0.012** | 0.198 | **0.018** | -0.322 | **< 0.001** |
| EF | -0.231 | **0.006** | 0.088 | 0.376 | -0.306 | **0.002** |
| HV | -0.037 | 0.564 | 0.200 | **0.006** | -0.184 | **0.011** |
| EC thickness | -0.108 | 0.098 | 0.185 | **0.014** | -0.217 | **0.004** |
| Ventricular volume | 0.416 | **0.042** | -0.652 | **0.006** | 0.889 | **< 0.001** |
