# Supplementary material for: Association of peripheral immunity with cognition, neuroimaging, and Alzheimer’s pathology
Source: Alzheimers Res Ther. 2022 Feb 9;14:29. doi: 10.1186/s13195-022-00968-y (PMC8830026; doi:10.1186/s13195-022-00968-y)
Supplement: Supplementary file 3 — Additional file 3. Longitudinal associations of peripheral immunity with cognition, neuroimaging and AD pathology in all participants. [file 13195_2022_968_MOESM3_ESM.docx]

| Variable | NEU | | LYM | | NLR | |
| --- | --- | --- | --- | --- | --- | --- |
|  | β | P | β | P | β | P |
| Aβ  P-tau | 0.072  -0.014 | 0.326  0.482 | -0.047  -0.025 | 0.575  0.401 | 0.102  -0.018 | 0.248  0.573 |
| T-tau | 0.037 | 0.534 | -0.018 | 0.794 | 0.043 | 0.549 |
| FDG-PET | -0.004 | 0.141 | 0.001 | 0.832 | -0.004 | 0.214 |
| MMSE | -0.037 | **0.005** | 0.058 | **< 0.001** | -0.081 | **< 0.001** |
| CDRSB | 0.023 | **< 0.001** | -0.026 | **< 0.001** | 0.038 | **< 0.001** |
| ADAS | 0.027 | **0.032** | -0.025 | 0.079 | 0.047 | **0.001** |
| MEM | -0.036 | **0.003** | 0.017 | 0.215 | -0.043 | **0.003** |
| EF | -0.057 | **0.011** | 0.007 | 0.780 | -0.055 | **0.034** |
| HV | -0.013 | 0.122 | 0.026 | **0.005** | -0.030 | **0.002** |
| EC thickness | -0.009 | 0.479 | 0.029 | **0.036** | -0.027 | 0.053 |
| ventricular volume | 0.034 | **0.032** | -0.038 | 0.055 | 0.057 | **0.002** |
