# Supplementary material for: Association of peripheral immunity with cognition, neuroimaging, and Alzheimer’s pathology
Source: Alzheimers Res Ther. 2022 Feb 9;14:29. doi: 10.1186/s13195-022-00968-y (PMC8830026; doi:10.1186/s13195-022-00968-y)
Supplement: Supplementary file 4 — Additional file 4. Cross-sectional associations of peripheral immunity with cognition, neuroimaging and AD pathology in CN group. [file 13195_2022_968_MOESM4_ESM.docx]

| Variable | NEU | | LYM | | NLR | |
| --- | --- | --- | --- | --- | --- | --- |
|  | Β | P | β | P | β | P |
| Aβ | -0.135  0.010 | 0.443  0.916 | 0.333  -0.017 | **0.020**  0.826 | -0.293  -0.003 | **0.030**  0.968 |
| P-tau |  |  |  |  |  |  |
| T-tau | 0.046 | 0.573 | -0.054 | 0.413 | 0.045 | 0.474 |
| FDG-PET | 0.010 | 0.692 | -0.016 | 0.448 | 0.015 | 0.445 |
| MMSE | 0.393 | **0.037** | -0.038 | 0.805 | 0.275 | 0.059 |
| CDRSB | 0.001 | 0.378 | 0.001 | 0.284 | 0.0001 | 0.924 |
| ADAS | -0.193 | 0.444 | -0.053 | 0.800 | -0.065 | 0.739 |
| MEM | -0.006 | 0.738 | 0.002 | 0.907 | -0.008 | 0.668 |
| EF | 0.096 | 0.414 | -0.073 | 0.604 | 0.103 | 0.442 |
| HV | 0.173 | 0.370 | -0.033 | 0.832 | 0.110 | 0.456 |
| EC thickness | -0.055 | 0.769 | -0.043 | 0.778 | 0.030 | 0.834 |
| ventricular volume | 0.066 | 0.408 | -0.104 | 0.117 | 0.132 | **0.037** |
