# Supplementary material for: Association of peripheral immunity with cognition, neuroimaging, and Alzheimer’s pathology
Source: Alzheimers Res Ther. 2022 Feb 9;14:29. doi: 10.1186/s13195-022-00968-y (PMC8830026; doi:10.1186/s13195-022-00968-y)
Supplement: Supplementary file 5 — Additional file 5. Cross-sectional associations of peripheral immunity with cognition, neuroimaging and AD pathology in MCI group. [file 13195_2022_968_MOESM5_ESM.docx]

| Variable | NEU | | LYM | | NLR | |
| --- | --- | --- | --- | --- | --- | --- |
|  | β | P | β | P | β | P |
| Aβ | -0.035 | 0.770 | 0.180 | **0.034** | -0.167 | 0.059 |
| P-tau | -0.006 | 0.934 | -0.069 | 0.172 | 0.036 | 0.494 |
| T-tau | -0.050 | 0.390 | -0.111 | **0.025** | 0.026 | 0.611 |
| FDG-PET | -0.070 | **< 0.001** | 0.003 | 0.838 | -0.043 | **0.003** |
| MMSE | -0.074 | 0.581 | -0.016 | 0.866 | 0.086 | 0.396 |
| CDRSB | 0.123 | **<0.001** | 0.011 | 0.623 | 0.052 | **0.021** |
| ADAS | 0.245 | 0.287 | -0.208 | 0.210 | 0.308 | 0.074 |
| MEM | -0.009 | 0.623 | 0.041 | **0.045** | -0.031 | 0.145 |
| EF | -0.168 | 0.084 | -0.007 | 0.950 | -0.143 | 0.222 |
| HV | 0.007 | 0.958 | 0.188 | **0.050** | -0.138 | 0.156 |
| EC thickness | -0.046 | 0.745 | 0.129 | 0.216 | -0.102 | 0.330 |
| ventricular volume | 0.282 | 0.460 | -0.278 | 0.314 | 0.358 | 0.211 |
