# Supplementary material for: Association of peripheral immunity with cognition, neuroimaging, and Alzheimer’s pathology
Source: Alzheimers Res Ther. 2022 Feb 9;14:29. doi: 10.1186/s13195-022-00968-y (PMC8830026; doi:10.1186/s13195-022-00968-y)
Supplement: Supplementary file 6 — Additional file 6. Cross-sectional associations of peripheral immunity with cognition, neuroimaging and AD pathology in AD group. [file 13195_2022_968_MOESM6_ESM.docx]

| Variable | NEU | | LYM | | NLR | |
| --- | --- | --- | --- | --- | --- | --- |
|  | β | P | β | P | β | P |
| Aβ | 0.072 | 0.554 | 0.064 | 0.772 | 0.121 | 0.498 |
| P-tau | -0.053 | 0.306 | 0.075 | 0.443 | -0.090 | 0.243 |
| T-tau | -0.261 | **0.038** | -0.131 | 0.580 | -0.139 | 0.464 |
| FDG-PET | 0.022 | 0.213 | 0.040 | 0.210 | 0.006 | 0.825 |
| MMSE | -0.078 | 0.535 | 0.045 | 0.845 | -0.090 | 0.624 |
| CDRSB | 0.004 | 0.921 | -0.077 | 0.271 | 0.002 | 0.977 |
| ADAS | -0.024 | 0.492 | -0.024 | 0.700 | -0.008 | 0.879 |
| MEM | 0.011 | 0.915 | 0.064 | 0.581 | -0.078 | 0.554 |
| EF | -0.007 | 0.901 | 0.025 | 0.710 | -0.024 | 0.746 |
| HV | -0.003 | 0.979 | 0.033 | 0.372 | -0.024 | 0.413 |
| EC thickness | -0.084 | 0.524 | 0.451 | 0.058 | -0.334 | 0.073 |
| ventricular volume | 0.028 | 0.567 | -0.122 | 0.167 | 0.081 | 0.254 |
