# Supplementary material for: Association of peripheral immunity with cognition, neuroimaging, and Alzheimer’s pathology
Source: Alzheimers Res Ther. 2022 Feb 9;14:29. doi: 10.1186/s13195-022-00968-y (PMC8830026; doi:10.1186/s13195-022-00968-y)
Supplement: Supplementary file 7 — Additional file 7. Longitudinal associations of peripheral immunity with cognition, neuroimaging and AD pathology in CN group. [file 13195_2022_968_MOESM7_ESM.docx]

| Variable | NEU | | LYM | | NLR | |
| --- | --- | --- | --- | --- | --- | --- |
|  | β | P | β | P | β | P |
| Aβ | -0.051 | 0.570 | 0.474 | **<0.001** | -0.291 | **0.011** |
| P-tau | -0.043 | 0.066 | -0.011 | 0.707 | -0.033 | 0.251 |
| T-tau | -0.073 | 0.688 | -0.071 | 0.760 | -0.006 | 0.980 |
| FDG-PET | -0.008 | 0.058 | -0.001 | 0.833 | -0.006 | 0.218 |
| MMSE | -0.020 | 0.332 | 0.023 | 0.343 | -0.026 | 0.289 |
| CDRSB | 0.003 | 0.122 | -0.007 | **0.008** | 0.007 | **0.004** |
| ADAS | 0.018 | 0.267 | -0.0002 | 0.992 | 0.007 | 0.727 |
| MEM | -0.016 | 0.318 | -0.002 | 0.902 | 0.002 | 0.913 |
| EF | -0.034 | 0.056 | 0.003 | 0.885 | -0.010 | 0.606 |
| HV | -0.011 | 0.483 | 0.040 | **0.051** | -0.042 | **0.027** |
| EC thickness | 0.015 | 0.442 | 0.023 | 0.381 | -0.003 | 0.888 |
| ventricular volume | -0.001 | 0.926 | -0.015 | 0.245 | 0.016 | 0.261 |
