# Supplementary material for: Association of peripheral immunity with cognition, neuroimaging, and Alzheimer’s pathology
Source: Alzheimers Res Ther. 2022 Feb 9;14:29. doi: 10.1186/s13195-022-00968-y (PMC8830026; doi:10.1186/s13195-022-00968-y)
Supplement: Supplementary file 8 — Additional file 8. Longitudinal associations of peripheral immunity with cognition, neuroimaging and AD pathology in MCI group. [file 13195_2022_968_MOESM8_ESM.docx]

| Variable | NEU | | LYM | | NLR | |
| --- | --- | --- | --- | --- | --- | --- |
|  | β | P | β | P | β | P |
| Aβ | 0.057 | 0.832 | 0.624 | **0.039** | -0.572 | 0.088 |
| P-tau | 0.007 | 0.807 | -0.009 | 0.781 | 0.031 | 0.409 |
| T-tau | 0.065 | 0.364 | -0.052 | 0.501 | 0.115 | 0.196 |
| FDG-PET | -0.002 | 0.488 | 0.001 | 0.805 | -0.006 | 0.218 |
| MMSE | 0.039 | 0.060 | 0.068 | **0.002** | -0.104 | **< 0.001** |
| CDRSB | 0.026 | **0.007** | -0.033 | **0.002** | 0.046 | **< 0.001** |
| ADAS | 0.019 | 0.433 | -0.045 | 0.090 | 0.064 | **0.024** |
| MEM | -0.037 | **0.028** | 0.018 | 0.316 | -0.052 | **0.010** |
| EF | -0.035 | 0.214 | -0.003 | 0.924 | -0.048 | 0.143 |
| HV | -0.011 | 0.328 | 0.027 | **0.026** | -0.026 | **0.039** |
| EC thickness | -0.024 | 0.159 | 0.035 | **0.050** | -0.039 | **0.038** |
| ventricular volume | 0.039 | 0.116 | -0.032 | 0.297 | 0.045 | 0.103 |
