# Supplementary material for: Association of peripheral immunity with cognition, neuroimaging, and Alzheimer’s pathology
Source: Alzheimers Res Ther. 2022 Feb 9;14:29. doi: 10.1186/s13195-022-00968-y (PMC8830026; doi:10.1186/s13195-022-00968-y)
Supplement: Supplementary file 9 — Additional file 9. Longitudinal associations of peripheral immunity with cognition, neuroimaging and AD pathology in AD group. [file 13195_2022_968_MOESM9_ESM.docx]

| Variable | NEU | | LYM | | NLR | |
| --- | --- | --- | --- | --- | --- | --- |
|  | β | P | β | P | β | P |
| Aβ | 0.206 | 0.233 | -0.135 | 0.483 | 0.406 | **0.050** |
| P-tau | 0.100 | 0.098 | 0.087 | 0.883 | -0.488 | 0.427 |
| T-tau | 0.037 | 0.318 | 0.041 | 0.279 | -0.012 | 0.771 |
| FDG-PET | 0.004 | 0.724 | 0.007 | 0.629 | 0.011 | 0.456 |
| MMSE | -0.014 | 0.896 | 0.070 | 0.535 | 0.057 | 0.629 |
| CDRSB | 0.007 | 0.702 | -0.026 | 0.188 | 0.046 | **0.025** |
| ADAS | 0.018 | 0.494 | -0.029 | 0.328 | 0.041 | 0.176 |
| MEM | 0.069 | 0.227 | 0.070 | 0.298 | 0.020 | 0.773 |
| EF | 0.019 | 0.884 | 0.107 | 0.345 | -0.042 | 0.728 |
| HV | 0.007 | 0.366 | 0.001 | 0.916 | 0.005 | 0.619 |
| EC thickness | 0.093 | 0.244 | -0.039 | 0.671 | 0.051 | 0.581 |
| ventricular volume | -0.203 | 0.528 | 0.018 | 0.810 | -0.039 | 0.587 |
