# Supplementary material for: Association of peripheral immunity with cognition, neuroimaging, and Alzheimer’s pathology
Source: Alzheimers Res Ther. 2022 Feb 9;14:29. doi: 10.1186/s13195-022-00968-y (PMC8830026; doi:10.1186/s13195-022-00968-y)
Supplement: Supplementary file 10 — Additional file 10. Interaction analysis. [file 13195_2022_968_MOESM10_ESM.docx]

|  | gender | | *APOE4* | |
| --- | --- | --- | --- | --- |
|  | **β** | P | **β** | P |
| CDRSB | -0.030 | 0.600 | -0.057 | 0.179 |
| ADAS | -0.104 | 0.537 | -0.062 | 0.617 |
| MEM | 0.070 | 0.617 | 0.032 | 0.759 |
| EF | 0.145 | 0.382 | 0.122 | 0.317 |
| FDG-PET | 0.019 | 0.449 | 0.015 | 0.415 |
| Ventricular volume | -0.538 | 0.187 | 0.440 | 0.144 |

|  | SEX | | *APOE4* | |
| --- | --- | --- | --- | --- |
|  | **β** | P | **β** | P |
| Aβ | -0.001 | 0.989 | -0.002 | 0.943 |
| T-tau | 0.046 | 0.538 | 0.023 | 0.676 |
| ADAS | 0.090 | 0.642 | -0.178 | 0.203 |
| MEM | -0.168 | 0.319 | 0.128 | 0.308 |
| HV | -0.082 | 0.570 | 0.077 | 0.456 |
| ventricular volume | 0.044 | 0.721 | -0.083 | 0.339 |
| EC thickness | -0.087 | 0.558 | 0.165 | 0.123 |

|  | SEX | | *APOE4* | |
| --- | --- | --- | --- | --- |
|  | **β** | P | **β** | P |
| Aβ | -0.024 | 0.540 | 0.018 | 0.525 |
| T-tau | -0.065 | 0.388 | -0.043 | 0.442 |
| CDRSB | 0.052 | 0.436 | 0.030 | 0.544 |
| ADAS | -0.057 | 0.769 | 0.049 | 0.731 |
| MEM | 0.170 | 0.383 | -0.073 | 0.541 |
| EF | 0.170 | 0.383 | 0.008 | 0.957 |
| FDG-PET | 0.030 | 0.316 | 0.008 | 0.701 |
| HV | 0.320 | **0.024** | 0.017 | 0.870 |
| Ventricular volume | -0.113 | 0.355 | 0.101 | 0.252 |
| EC thickness | 0.099 | 0.501 | -0.187 | 0.085 |
